# Editorial: Searching for meaning in work and life: happiness, wellbeing and the future of organizations

**DOI:** 10.3389/fpsyg.2023.1287404

**Published:** 2023-09-25

**Authors:** Michela Floris, Lucrezia Casulli, Laura Ferrari

**Affiliations:** ^1^Department of Economics and Business Sciences, University of Cagliari, Cagliari, Italy; ^2^Hunter Centre for Entrepreneurship, University of Strathclyde, Glasgow, United Kingdom; ^3^Catholic University of Milan, Family Studies and Research University Centre, Milan, Italy

**Keywords:** meaning, happiness, wellbeing, work-place, organization

Finding meaning in one's work and in one's life is a quest that has a powerful resonance in an era that values holistic fulfillment. This Research Topic invited cross-disciplinary contributions to shed light on the future of organizations seen through a meaning-making lens of employment across a number of different organizational contexts. We received a series of insightful research pieces that untangle the web of connections between happiness, wellbeing, and the workings of organizations.

Starting with a provocative stance, Pelligra and Sacco postulation is that, in a post-scarcity society, where people are increasingly looking for meaning in the workplace, meaning and purpose are increasingly eluding work in ever more complex organizations. Drawing on anthropologist David Graeber's notion of “bullshit jobs,” the authors identify the root causes of meaningless, unsatisfying work in the design and conditions of many of today's jobs. The design of many jobs is such that individuals cannot see the value they are adding to society, and they themselves come to consider their employment useless to society and, thus, meaningless. This has significant repercussions on people's wellbeing. The authors point out that the economic argument for compensating workers for dis-utility has seriously underestimated the more complex psychological needs of human beings for self-worth, trust, and agency in the workplace. On the flip side, they warn against the risk of burnout for those whose jobs are so meaningful that they can give too much of themselves, especially those creating social value.

For the skeptics out there, who see the world as a zero-sum game, Parent-Lamarche et al. offer reassurance that HRM practices do not have to exist in order to squeeze every last drop of productivity out of employees at the expense of employee wellbeing. Their validated scale of High Wellbeing and Performance HRM practices, firmly grounded in an integrated mutual gains perspective (i.e., non-zero-sum games), provides a viable model for promoting employee wellbeing, thus leading to productivity. Their scale validation distills 10 dimensions of HRM practices, including autonomy, on-the-job training (formation), opportunities for career progression (career management), equality, diversity, and inclusion (diversity management), and flexible work (flexibility), to highlight but a few.

The study by Bryant et al. on the idea of “contributing to society” explores the foundation of satisfying employment and vocation. This crucial idea is considered important, yet it is still buried in much mystery. The authors reinterpret it as a profound sensation of positively affecting people. The Situated Expectancy–Value Theory, which reveals **three** dimensions of fulfillment—matching with one's purpose, investing personally, aligning contributions with expectations, and measuring the accompanying costs—is the foundation of their ground-breaking perspective. Like a prism, this paradigm highlights the rewarding echoes within oneself while refracting task value dependent on beneficial circumstances. This innovative strategy opens up new research directions in meaningful work, social effect, and areas such as employment design and public policy.

Su and Jiang research digs into the complex interplay of work–family dynamics among Chinese female university lecturers, focusing on their conventional responsibilities. They use latent variable path analysis with 527 participants to show that work–family conflicts and burnout have a negative impact on job satisfaction. In contrast, perceived organizational support has a favorable effect. To some extent, job burnout mediates the relationship between conflicts and job satisfaction, with perceived organizational support reducing this mediation. This study sheds insight into the complicated dynamics of female university professors balancing work, family, burnout, support, and job satisfaction within the cultural environment of China.

In pursuing an optimal state of being, Clapp et al. research breathes life into the elusive concept of psychological flow. This delicate equilibrium, with its interplay of task challenge and skill mastery, creates an intrinsic symphony of satisfaction. The study unveils the elements composing this symphony, charting their evolution in professional and leisure domains. With insights gathered through candid semi-structured interviews with transactional workers, the study highlights the flow experience within constrained roles. The discovery of two primary flow types adds depth to the narrative, further enriched as traditional flow dimensions harmonize with participants' personal experiences.

Thompson exploration navigates the realm of awe's impact on resilience and wellbeing. The study's focus on a NASA medical professional—a leader of both minds and hearts—reverberates significantly. The intricate dance between awe and resilience, as navigated by this professional amid their role supporting astronauts, opens a window into personal and professional transformation. As awe unfolds its transformative magic, it reveals itself as an elixir for uncovering life's purpose, sowing seeds of gratitude, weaving bonds of connection, and nurturing resilience traits that sustain optimism. This research highlights the ethereal yet tangible threads intertwining awe, resilience, and personal evolution.

Smaliukiené et al. study takes us into the unique realm of military service—defined by unwavering dedication to one's nation and the noble aspiration to uplift others. A chorus of duty echoes among the ranks of army reservists, often straddling civilian obligations and military commitments. Recent research places the spotlight on the synergy between prosocial motivation and the profoundness of service. The study fills a void in scholarship, offering insights into the latent power of prosocial drive on the tapestry of service's significance. This research forges pathways of understanding, unveiling the mosaic where prosocial motivation intertwines with role fit, self-efficacy, and the socio-moral climate of military service.

These studies collectively weave a tapestry of insight into the often-oversimplified needs of human beings by sharpening our understanding of the convergence of meaning, happiness, and the dynamics of today's organizations. Notably, what emerges from these contributions taken together is a harmonious chorus of findings, all singing from the same hymn sheet: factors such as purpose, autonomy, and mutual support, amongst many others, are the golden thread across all the contributions. These factors are at the heart of thriving in a complex world—where work, family, and personal journey intermingle, creating a symphony of resilience, fulfillment, and flourishing.

## Author contributions

MF: Writing—original draft, Writing—review and editing. LC: Writing—original draft, Writing—review and editing. LF: Writing—original draft, Writing—review and editing.

